# Numerical modelling of acoustic cavitation threshold in water with non-condensable bubble nuclei

**DOI:** 10.1016/j.ultsonch.2022.105932

**Published:** 2022-01-29

**Authors:** Seongjin Hong, Gihun Son

**Affiliations:** Department of Mechanical Engineering, Sogang University, 35 Baekbeom-ro, Mapo-gu, Seoul 04107, South Korea

**Keywords:** Cavitation, Heat and mass transfer, Nanobubble, Threshold, Ultrasound

## Abstract

Numerical modelling of acoustic cavitation threshold in water is presented taking into account non-condensable bubble nuclei, which are composed of water vapor and non-condensable air. The cavitation bubble growth and collapse dynamics are modeled by solving the Rayleigh-Plesset or Keller-Miksis equation, which is combined with the energy equations for both the bubble and liquid domains, and directly evaluating the phase-change rate from the liquid and bubble side temperature gradients. The present work focuses on elucidating acoustic cavitation in water with a wide range of cavitation thresholds (0.02–30 MPa) reported in the literature. Computations for different nucleus sizes and acoustic frequencies are performed to investigate their effects on bubble growth and cavitation threshold. The numerical predictions are observed to be comparable to the experimental data in the previous works and show that the cavitation threshold in water has a wide range depending on the bubble nucleus size.

## Highlights

• A numerical model is presented for acoustic cavitation in water with non-condensable bubble nuclei.
• The phase change has a significant effect on bubble growth and collapse dynamics during acoustic cavitation.
• As the bubble nucleus size increases and the acoustic frequency increases, the cavitation threshold increases beyond the Blake threshold.
• The threshold predictions fitted as a function of bubble nucleus size and acoustic frequency can be applied to acoustic cavitation in water with a wide range of threshold data in the previous works.

## Nomenclature

a,b: constants in the Van der Waals equation ($m^{6}\mathrm{Pa}/{\mathrm{kg}}^{2},m^{3}/\mathrm{kg}$)
C: speed of sound (m/s)
c: specific heat (J/kgK)
D: diffusion coefficient ($m^{2}/s$)
e: specific energy (J/kg)
f: acoustic frequency (Hz)
G: phase-change mass flux ($\mathrm{kg}/m^{2}s$)
$h_{\mathrm{fg}}$: latent heat of vaporization (J/kg)
M: molar mass (kg/kmol)
p: pressure (Pa)
$p_{A}$: acoustic amplitude (Pa)
$p_{B}$: Blake threshold (Pa)
$p_{\mathrm{th}}$: cavitation threshold (Pa)
Y: air mass fraction
$R_{b}$: bubble radius (m)
$R_{g}$: gas constant (J/kgK)
r: spherical coordinate (m)
T: temperature (K)

**Greek symbols**
α: accommodation coefficient
μ: viscosity (Pas)
λ: thermal conductivity (W/mK)
σ: surface tension (N/m)
ρ: density ($\mathrm{kg}/m^{3}$)

**Subscripts**
a,v: air, vapor
av: average
b,l: bubble, liquid
cr: critical
o: initial
s: bubble-liquid interface
sat: saturation
∞: ambient

## 1 Introduction

Cavitation is a vapor generation process in a liquid under the local saturation pressure [1]. When cavitation occurs due to intense ultrasound pulses, the generated bubbles expand and experience rapid collapse, which is called acoustic cavitation. When the bubble collapses, it is greatly compressed and heated to a high temperature, releasing enormous energy into the surrounding fluid [2]. The acoustic cavitation has been extensively studied for many engineering applications including water treatment [3], medical therapy [4] and surface cleaning [5], as reviewed in Ref. [2]. However, a general predictive model for the threshold of acoustic cavitation in water, which has been measured over a wide range of 0.02–30 MPa [6], [7], [8], [9], [10], is lacking in the literature.

Cavitation is divided into two categories depending on its inception: homogeneous cavitation of vapor bubbles in a metastable pure liquid [11] and heterogeneous cavitation due to impurities such as solid particles and non-condensable gas nuclei [12]. Recent experimental studies have shown that bulk nanobubbles with a radius of less than 500nm exist in ambient water and survive for more than a few weeks [13]. The nanobubbles have great potential in acoustic cavitation because they act as heterogeneous cavitation nuclei and significantly reduce the cavitation threshold than the homogeneous bubble cavitation case. However, their quantitative effect on the cavitation threshold has been rarely reported in the literature.

The cavitation threshold has been estimated theoretically by two models based on vapor bubble nucleation or large growth of pre-existing bubbles. Classical nucleation theory (CNT) [11] is a popular model that describes homogeneous cavitation of bubble nuclei in a pure liquid. The prediction of CNT for cavitation threshold is higher than 100 MPa in standard water. However, this value is very different from the cavitation threshold below 30 MPa measured in typical experiments [6], [7], [8], [9], [10]. In heterogeneous cavitation from pre-existing bubble nuclei, the Blake threshold [14] is a useful concept for predicting the minimum liquid pressure that causes explosive growth of bubble nuclei. The minimum pressure can be evaluated by combining the bubble nucleus size, surface tension and vapor pressure. However, the Blake threshold concept does not take into account the temporal influence of acoustic frequency, which causes a deviation from the acoustic cavitation threshold at ultrasonic frequencies of a few MHz [15].

Analytical and numerical models for predicting the acoustic cavitation threshold have been developed in several studies. Holland and Apfel [15] presented an analytical approach for the cavitation threshold of air bubbles in water over various acoustic frequencies of 0.5–10 MHz and initial bubble radii of 0.1–3 μm. Assuming that the bubble follows an adiabatic process and using the cavitation criterion that the maximum collapse temperature exceeds 5000 K, causing free radical production. they observed that when the initial bubble radius was 0.7μm, the cavitation threshold increased linearly with acoustic frequency and minimized to 0.28MPa at a frequency of 1MHz. Maxwell et al. [9] experimentally observed that the cavitation threshold in water is about 27MPa at an acoustic frequency of 1.1MHz. In their numerical model, cavitation was assumed to originate from a spherical gas nucleus with a radius of 2.5 nm. When the cavitation threshold was defined as the condition that the maximum bubble radius is $10^{4}$ times larger than the initial radius, the predicted threshold of 28.1 MPa was comparable to the experimental observation. Suo et al. [16] also conducted a similar analysis to explore the influence of multi-frequency ultrasound on the microbubble cavitation threshold using two criteria based on the maximum bubble radius larger than twice the initial radius and the collapse rate larger than the speed of sound.

Although cavitation represents a phase-change phenomenon with bubble generation, the previous analytical and numerical models for acoustic cavitation have often neglected the phase-change effect [9], [15], [16], [17], [18], [19]. The growth and collapse of gas bubbles without phase change, which is called gaseous cavitation (or pseudo cavitation) [20], differs from actual cavitation [21]. The influences of heat transfer and phase change on cavitation bubble growth and collapse were investigated earlier by Fujikawa and Akamatsu [22] considering the collapse of a bubble with initial radii of 0.1–1 mm. The bubble collapse rate was observed to be slightly lower in the case with heat transfer and vapor condensation than in the adiabatic case. Yasui [23] studies the effects of thermal conduction and phase change on acoustic cavitation of air-vapor mixture bubbles with initial radii of 4.5–10.5 μm at an acoustic frequency of 26.5kHz and acoustic amplitudes of 1–1.275 atm. The numerical results were observed to match with the experimental data including thermal conduction than the case without thermal conduction. Using similar acoustic and bubble conditions, Storey and Szeri [24] performed a systematic analysis to demonstrate the influences of phase change and chemical reaction on collapsing bubble dynamics. They showed that excess water vapor is trapped in the collapsing bubble and significantly reduces the bubble peak temperature. Recently, Peng et al. [25] conducted a similar analysis for acoustic cavitation of vapor and argon mixture bubbles with initial radii of 1.5 and 4.5 μm and obtained the optimum liquid temperature that maximizes the bubble collapse intensity, depending on the acoustic frequency and amplitude. Dehane et al. [26], [27], [28], [29] also performed a numerical analysis for acoustic cavitation of ambient (or initial) bubbles with radii of 0.5–14 μm, including the effects of heat and mass transfer and chemical reaction. They investigated the bubble collapse temperature and pressure in various acoustic amplitude and frequency conditions. The effects of thermal conduction and mass transfer were observed to be dominant mechanisms depending on bubble size and acoustic amplitude. The chemical reaction had an insignificant influence on the maximum bubble radius, but had a tendency to lower the maximum bubble temperature due to the endothermal chemical reactions occurring within the bubble.

Although the previous works [23], [24], [25], [26], [27], [28], [29] have advanced the analysis of acoustic cavitation including the phase-change effect, their analysis was limited in that the heat and mass transfer rates were calculated from the boundary layer approximations with adjusting factors instead of solving the conservation equations. Their applications were mainly acoustic cavitation of microbubbles, which are relatively larger than the nanobubble nuclei [13] expected in real tap or degassed water. Few studies have applied such a model to predict the wide range of acoustic cavitation thresholds (0.02–30 MPa) measured in typical experiments [6], [7], [8], [9], [10].

In this work, a general numerical modelling of acoustic cavitation in water is developed by combining the Rayleigh-Plesset (RP) or Keller-Miksis (KM) equation with the energy equations for both the bubble and liquid domains and directly evaluating the phase-change rate from the liquid and bubble side temperature gradients. The numerical model is applied to acoustic cavitation in water with non-condensable bubble nuclei to clarify a broad range of cavitation thresholds reported in the literature. We consider nanobubbles with a radius of less than 500nm, which are known to exist in degassed or tap water and survive for a few weeks [13], and acoustic frequencies of 0.1–5 MHz, which are used in many engineering applications [2] including sonochemistry, water treatment and surface cleaning. Various acoustic amplitudes are tested to find the cavitation threshold depending on the bubble nucleus size and acoustic frequency.

## 2 Numerical analysis

The current analysis focuses on acoustic cavitation in water with a non-condensable bubble nucleus, which is assumed to be a sphere with a radius of $R_{\mathrm{bo}}$ and a mixture of non-condensable air and water vapor. It is initially in mechanical and thermal equilibrium with the ambient water at 1 atm and 293 K and then grows or collapses as an acoustic pulse is applied. Water is considered an incompressible fluid, whereas the bubble is treated a Van der Waals (VDW) gas to describe the high-pressure state during collapse. The equation of state (EOS) is written as

(1) $$p=\frac{\rho R_{g}T}{1-b\rho }-a\rho^{2}$$

where $R_{g},a$ and *b* are the gas constant and VDW constants. For a mixture bubble, the constants are determined using the air mass fraction *Y* of the bubble as [23]

(2) $$a_{b}=a_{a}Y^{2}+2\sqrt{a_{a}a_{v}}Y(1-Y)+a_{v}{\left(1-Y\right)}^{2}$$

(3) $$b_{b}=b_{a}\frac{M_{b}}{M_{a}}Y^{2}+\frac{M_{b}}{4}{\left(\frac{b_{a}^{1/3}M_{a}^{1/3}+b_{v}^{1/3}M_{v}^{1/3}}{M_{a}^{1/3}M_{v}^{1/3}}\right)}^{3}Y(1-Y)+b_{v}\frac{M_{b}}{M_{v}}{\left(1-Y\right)}^{2}$$

Here, the subscripts a,v and *b* denote air, vapor and bubble, respectively. The mixture molar mass $M_{b}$ is calculated as $M_{b}={\left[Y/M_{a}+(1-Y)/M_{v}\right]}^{-1}$.

### 2.1 Governing equations

The conservation equations of mass, air mass fraction *Y*, momentum and energy in the spherical bubble region are written as

(4) $$\frac{\partial r^{2}\rho_{b}}{\partial t}=-\frac{\partial }{\partial r}(r^{2}\rho_{b}u_{b})$$

(5) $$\frac{\partial r^{2}\rho_{b}Y}{\partial t}=-\frac{\partial }{\partial r}(r^{2}\rho_{b}u_{b}Y)+\frac{\partial }{\partial r}(r^{2}\rho_{b}D\frac{\partial Y}{\partial r})$$

(6) $$\frac{\partial r^{2}\rho_{b}u_{b}}{\partial t}=-\frac{\partial }{\partial r}(r^{2}\rho_{b}u_{b}^{2})-r^{2}\frac{\partial p_{b}}{\partial r}+\frac{4}{3}[\frac{\partial }{\partial r}r^{2}\mu_{b}(\frac{\partial u_{b}}{\partial r}-\frac{u_{b}}{r})+r\mu_{b}(\frac{\partial u_{b}}{\partial r}-\frac{u_{b}}{r})]$$

(7) $$\frac{\partial r^{2}\rho_{b}e_{b}}{\partial t}=-\frac{\partial }{\partial r}(r^{2}\rho_{b}u_{b}e_{b})-p_{b}\frac{\partial }{\partial r}(r^{2}u_{b})+\frac{\partial }{\partial r}r^{2}\lambda_{b}\frac{\partial T_{b}}{\partial r}+\frac{4}{3}r^{2}\mu_{b}{\left(\frac{\partial u_{b}}{\partial r}-\frac{u_{b}}{r}\right)}^{2}$$

Here, *D* is the diffusion coefficient, $\mu_{b}$ is the viscosity and $\lambda_{b}$ is the thermal conductivity. The temperature and pressure are calculated from $T_{b}=(e_{b}+a_{b}\rho_{b})/c_{b}$ and the VDW Eq. (1), where $c_{b}$ is the specific heat evaluated as $c_{b}=c_{a}Y+c_{v}(1-Y)$.

Assuming that the liquid is incompressible and contains no dissolved gases, the conservation equations in the liquid region are written as

(8) $$\frac{1}{r^{2}}\frac{\partial }{\partial r}(r^{2}u_{l})=0$$

(9) $$\rho_{l}(\frac{\partial u_{l}}{\partial t}+u_{l}\frac{\partial u_{l}}{\partial r})=-\frac{\partial p_{l}}{\partial r}+\mu_{l}[\frac{1}{r^{2}}\frac{\partial }{\partial r}(r^{2}\frac{\partial u_{l}}{\partial r})-\frac{2u_{l}}{r^{2}}]$$

(10) $${\left(\rho c\right)}_{l}(\frac{\partial T_{l}}{\partial t}+u_{l}\frac{\partial T_{l}}{\partial r})=\frac{1}{r^{2}}\frac{\partial }{\partial r}(r^{2}\lambda_{l}\frac{\partial T_{l}}{\partial r})+2\mu_{l}[{\left(\frac{\partial u_{l}}{\partial r}\right)}^{2}+2{\left(\frac{u_{l}}{r}\right)}^{2}]$$

The conservation equations in the bubble and liquid regions are coupled through the matching conditions at $r=R_{b}$,

(11) $$\rho_{l}({\overset{˙}{R}}_{b}-u_{\mathrm{ls}})=\rho_{\mathrm{bs}}({\overset{˙}{R}}_{b}-u_{\mathrm{bs}})=G$$

(12) $$\rho_{\mathrm{bs}}(u_{\mathrm{bs}}-{\overset{˙}{R}}_{b})Y_{s}-\rho_{\mathrm{bs}}D\frac{\partial Y}{\partial r}=0$$

(13) $$p_{\mathrm{ls}}=p_{\mathrm{bs}}-\frac{2\sigma }{R_{b}}+G^{2}(\frac{1}{\rho_{\mathrm{bs}}}-\frac{1}{\rho_{l}})+2\mu_{l}\frac{\partial u_{l}}{\partial r}-\frac{4}{3}\mu_{b}(\frac{\partial u_{b}}{\partial r}-\frac{u_{b}}{r})$$

(14) $$T_{\mathrm{ls}}=T_{\mathrm{bs}}$$

Here, $\overset{˙}{R_{b}}={\mathrm{dR}}_{b}/\mathrm{dt}$ and *G* is the phase-change mass flux. Eq. (11) represents that the phase-change mass flux across the bubble surface is the same on the liquid and gas sides. In Eq. (12), non-condensable gas is assumed to have no mass flux across the the bubble surface. Eq. (13) indicates the force balance at the bubble surface including the effects of pressure difference, surface tension, phase change and viscous stresses. In Eq. (14), the temperature discontinuity at the bubble surface is neglected. Considering the energy balance at $r=R_{b}$, *G* is related to the liquid and bubble side heat fluxes as

(15) $$G=\frac{1}{h_{\mathrm{fg}}}[\lambda_{l}{\left(\frac{\partial T_{l}}{\partial r}\right)}_{r=R_{b}}-\lambda_{b}{\left(\frac{\partial T_{b}}{\partial r}\right)}_{r=R_{b}}]$$

Using the kinetic theory and assuming that the phase-change mass flux is low, *G* can be expressed as [22], [23], [24], [25], [26], [27], [28], [29], [30]

(16) $$G=\frac{2\alpha }{2-\alpha }\frac{p_{v,\mathrm{sat}}(T_{\mathrm{ls}})-p_{\mathrm{vs}}}{\sqrt{2\pi R_{g,v}T_{\mathrm{ls}}}}$$

where the accommodation coefficient α varies in the range of 0–0.35 depending on $T_{\mathrm{ls}}$ [25], [28], [31], the saturated vapor pressure $p_{v,\mathrm{sat}}$ at $T_{\mathrm{ls}}$ is determined by the Antoine Eq. (17), and the vapor pressure $p_{\mathrm{vs}}$ at the bubble surface, is calculated from the VDW Eq. (1) of water vapor using $\rho_{\mathrm{vs}}=\rho_{\mathrm{bs}}(1-Y_{s})$.

(17) $$p_{v,\mathrm{sat}}(T_{\mathrm{ls}})=\exp[23.3025-\frac{3893}{T_{\mathrm{ls}}-42.68}]$$

The bubble surface temperature $T_{\mathrm{ls}}$ can be iteratively determined by combining Eqs. (15), (16), (17).

The boundary conditions at r=∞ are described as

(18) $$u_{l}=0$$

(19) $$p_{l}=p_{\infty }+p_{\mathrm{ac}}$$

(20) $$T_{l}=T_{\infty }$$

where $p_{\mathrm{ac}}$ is an acoustic pressure.

The liquid velocity profile is solved from the mass Eq. (8) with the boundary conditions as

(21) $$u_{l}=u_{\mathrm{ls}}\frac{R_{b}^{2}}{r^{2}}$$

Integrating the momentum Eq. (9) over the liquid region and using the boundary conditions, we obtain the RP equation as

(22) $$\rho_{l}R_{b}\frac{{\mathrm{du}}_{\mathrm{ls}}}{\mathrm{dt}}=-2\rho_{l}u_{\mathrm{ls}}{\overset{˙}{R}}_{b}+\frac{1}{2}\rho_{l}u_{\mathrm{ls}}^{2}+p_{\mathrm{bs}}-p_{l}-\frac{2\sigma }{R_{b}}+G^{2}(\frac{1}{\rho_{\mathrm{bs}}}-\frac{1}{\rho_{l}})-4\mu_{l}\frac{u_{\mathrm{ls}}}{R_{b}}$$

### 2.2 Numerical methods

To efficiently treat the moving bubble surface, we introduce the moving coordinates $\xi_{b}=r/R_{b}$ for the bubble region and $\xi_{l}=r-R_{b}$ for the liquid region. The conservation Eqs. (4), (5), (6), (7) are rewritten as

(23) $$\frac{\partial r^{2}\rho_{b}}{\partial t}=-\frac{\partial }{\partial r}[r^{2}(u_{b}-\xi_{b}\overset{˙}{R_{b}})\rho_{b}]$$

(24) $$\frac{\partial r^{2}\rho_{b}Y}{\partial t}=-\frac{\partial }{\partial r}[r^{2}(u_{b}-\xi_{b}\overset{˙}{R_{b}})\rho_{b}Y]+\frac{\partial }{\partial r}(r^{2}\rho_{b}D\frac{\partial Y}{\partial r})$$

(25) $$\frac{\partial r^{2}\rho_{b}u_{b}}{\partial t}=-\frac{\partial }{\partial r}[r^{2}(u_{b}-\xi_{b}\overset{˙}{R_{b}})\rho_{b}u_{b}]-r^{2}\frac{\partial p_{b}}{\partial r}+\frac{4}{3}[\frac{\partial }{\partial r}r^{2}\mu_{b}(\frac{\partial u_{b}}{\partial r}-\frac{u_{b}}{r})+r\mu_{b}(\frac{\partial u_{b}}{\partial r}-\frac{u_{b}}{r})]$$

(26) $$\frac{\partial r^{2}\rho_{b}e_{b}}{\partial t}=-\frac{\partial }{\partial r}[r^{2}(u_{b}-\xi_{b}\overset{˙}{R_{b}})\rho_{b}e_{b}]-p_{b}\frac{\partial }{\partial r}(r^{2}u_{b})+\frac{\partial }{\partial r}r^{2}\lambda_{b}\frac{\partial T_{b}}{\partial r}+\frac{4}{3}r^{2}\mu_{b}{\left(\frac{\partial u_{b}}{\partial r}-\frac{u_{b}}{r}\right)}^{2}$$

(27) $${\left(\rho c\right)}_{l}(\frac{\partial T_{l}}{\partial t}+(u_{l}-\overset{˙}{R_{b}})\frac{\partial T_{l}}{\partial r})=\frac{1}{r^{2}}\frac{\partial }{\partial r}(r^{2}\lambda_{l}\frac{\partial T_{l}}{\partial r})+2\mu_{l}[{\left(\frac{\partial u_{l}}{\partial r}\right)}^{2}+2{\left(\frac{u_{l}}{r}\right)}^{2}]$$

The conservation equations are spatially discretized using a 2nd-order essentially non-oscillatory scheme [32], [33] for convection terms, and a central difference scheme for diffusion terms. The bubble region is discretized using 17–50 grid points, and the water region of $R_{b}<r⩽L$ is chosen to be large enough to exclude the influence of domain size, e.g. L>1m. A grid spacing of $\Delta r=R_{\mathrm{bo}}/10$ is used for $R_{b}<r<R_{b}+80R_{\mathrm{bo}}$, and non-uniform grids are used for the outer region. While introducing the moving coordinate $\xi_{l}=r-R_{b}$ for the liquid region, the outer range, $L=\xi_{l,L}+R_{b}$, changes with time. A 3rd-order total variation diminishing Runge–Kutta method [34] is employed to solve the transient differential equations in combination with an adaptive time-step algorithm that keeps the numerical errors estimated with two different time steps constant [35].

Eq. (22) and Eqs. (23), (24), (25), (26), (27) are solved for $u_{\mathrm{ls}},\rho_{b},Y,u_{b},e_{b}$ and $T_{l}$ with the matching and boundary conditions, given by Eqs. (11), (12), (14), (20), where $R_{b}$ and *G* is calculated by Eqs. (11), (15).

We consider a bubble nucleus composed of non-condensable air and water vapor at $p_{\infty }=1\mathrm{atm}$ and $T_{\infty }=293K$ and use the following water and air properties: $\rho_{l}=998\mathrm{kg}/m^{3},\mu_{l}=1\times 10^{-3}\mathrm{Pas},c_{l}=4.2\times 10^{3}J/\mathrm{kgK}$, $\lambda_{l}=0.6W/\mathrm{mK}$, $\mu_{v}=8.9\times 10^{-6}\mathrm{Pas},\mu_{a}=1.81\times 10^{-5}\mathrm{Pas}$, $c_{v}=1.4\times 10^{3}J/\mathrm{kgK},c_{a}=7.2\times 10^{2}J/\mathrm{kgK}$, $\lambda_{v}=1.9\times 10^{-2}W/\mathrm{mK},\lambda_{a}=2.5\times 10^{-2}W/\mathrm{mK}$, $R_{g,v}=461.5J/\mathrm{kgK},R_{g,a}=287J/\mathrm{kgK}$, $a_{v}=1727.2m^{6}\mathrm{Pa}/{\mathrm{kg}}^{2},a_{a}=166.7m^{6}\mathrm{Pa}/{\mathrm{kg}}^{2}$, $b_{v}=1.7\times 10^{-3}m^{3}/\mathrm{kg},b_{a}=1.3\times 10^{-3}m^{3}/\mathrm{kg}$, $h_{\mathrm{fg}}=2.45\times 10^{6}J/\mathrm{kg},T_{v,\mathrm{cr}}=652K$, $\sigma =7.30\times 10^{-2}N/m,D_{b}=2.6\times 10^{-5}m^{2}/s$. The mixture properties in the bubble are interpolated using the air mass fraction Y of the bubble as $c_{b}=c_{a}Y+c_{v}(1-Y),\lambda_{b}=\lambda_{a}Y+\lambda_{v}(1-Y)$, and $\mu_{b}=\mu_{a}Y+\mu_{v}(1-Y)$.

When the bubble temperature exceeds a critical value $T_{v,\mathrm{cr}}$ at bubble collapse and the thermodynamic difference between liquid water and vapor disappears [36], we assume no phase change (G=0), as done by Refs. [36], [37]. The bubble surface temperature $T_{\mathrm{bs}}$ is determined from the heat balance, $\lambda_{b}(\partial T_{b}/\partial r)=\lambda_{l}(\partial T_{l}/\partial r)$. When the bubble is at the supercritical state where the bubble surface velocity is above the speed of sound, the following Keller-Miksis equation [38] is solved instead of the RP equation to include the effect of liquid compressibility:

(28) $$\left(1-\frac{u_{\mathrm{ls}}}{C_{l}})R_{b}\frac{{\mathrm{du}}_{\mathrm{ls}}}{\mathrm{dt}}=-\frac{3}{2}u_{\mathrm{ls}}^{2}(1-\frac{u_{\mathrm{ls}}}{3C_{l}})+\frac{1}{\rho_{l}}(1+\frac{u_{\mathrm{ls}}}{C_{l}}+\frac{R_{b}}{C_{l}}\frac{d}{\mathrm{dt}})(p_{\mathrm{bs}}-p_{l}-\frac{2\sigma }{R_{b}}-4\mu_{l}\frac{u_{\mathrm{ls}}}{R_{b}}\right)$$

The following acoustic pressure pulse is imposed on ambient water:

(29) $$p_{\mathrm{ac}}=-p_{A}\sin(2\pi \mathrm{ft})$$

where $p_{A}$ and *f* represent the acoustic amplitude and frequency, respectively. The acoustic pulse is applied for two cycles (t⩽2/f) in the current computations.

## 3 Results and discussion

We choose a spherical bubble nucleus of $R_{\mathrm{bo}}=500\mathrm{nm}$ as a base case. The bubble is a mixture of air and water vapor, and is in mechanical and thermal equilibrium with the surrounding water at $p_{\infty }=1\mathrm{atm}$ and $T_{\infty }=293K$. The initial bubble pressure and temperature are evaluated as $p_{\mathrm{bo}}=p_{\infty }+2\sigma /R_{\mathrm{bo}}$ and $T_{\mathrm{bo}}=T_{\infty }$, respectively. The partial vapor pressure $p_{\mathrm{vo}}$ of the bubble can be determined from the Antoine Eq. (17). Using $\rho_{\mathrm{vo}}=\rho_{\mathrm{bo}}(1-Y_{o})$ and the VDW equations for air-vapor mixture and pure vapor, the initial air mass fraction of the bubble is iteratively obtained as $Y_{o}=0.996$.

### 3.1 Model validation

**Fig. 1:**
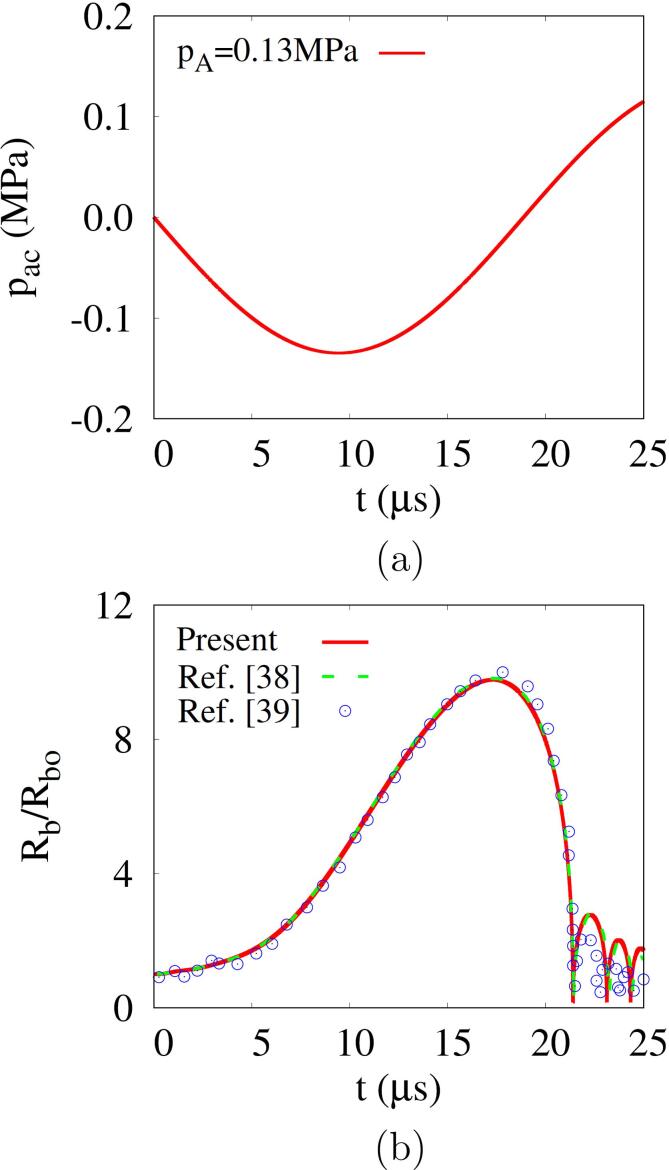
Validation of the present numerical model: (a) the acoustic pressure used in the calculation and (b) comparison of the predicted bubble radius with the previous numerical result [38] and experimental data [39].

To validate the present numerical model, computations are first carried out for the case of $R_{\mathrm{bo}}=4.5\mu m,p_{A}=0.13\mathrm{MPa}$ and f=26.5kHz, for which numerical results and experimental data are available in the literature [38], [39]. The results are plotted in Fig. 1. The initial bubble expands during the negative pressure cycle of acoustic pulse, reaching a maximum radius of $R_{b,\max}=9.8R_{\mathrm{bo}}$. As the acoustic pressure turns into a positive pulse, the bubble shrinks and collapses rapidly. The predicted bubble radius matches well with the previous numerical result [38] and experimental data [39]. The bubble temperature averaged over the bubble region ($0<r<R_{b}$) reaches $1.11\times 10^{4}K$ upon bubble collapse, which is similar to the previous numerical result of $1.10\times 10^{4}K$ [38].

### 3.2 Acoustic cavitation of an air-vapor mixture bubble nucleus

**Fig. 2:**
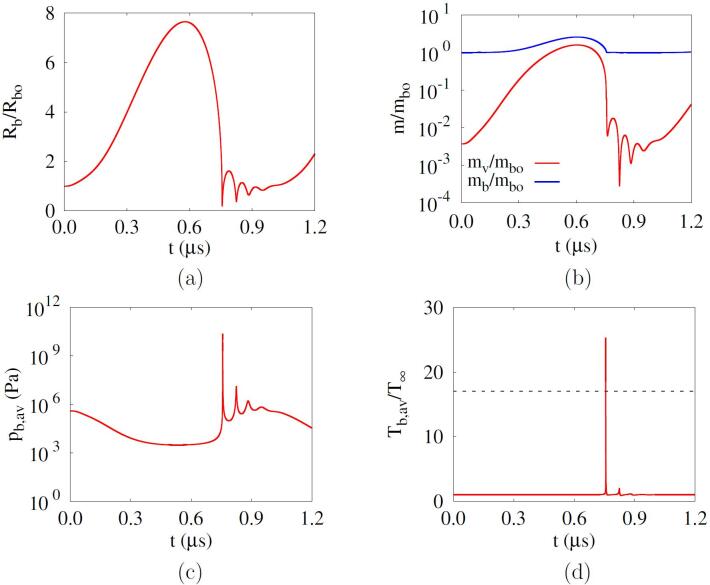
Acoustic cavitation of an air-vapor mixture bubble nucleus at $R_{\mathrm{bo}}=500\mathrm{nm},f=1\mathrm{MHz}$ and $p_{A}=0.35\mathrm{MPa}$: (a) bubble radius, (b) bubble mass, (c) bubble average pressure, (d) bubble average temperature. In the figure d, the dash line represents 5000K.

Fig. 2 shows the results of the phase-change cavitation at $R_{\mathrm{bo}}=500\mathrm{nm},f=1\mathrm{MHz}$ and $p_{A}=0.35\mathrm{MPa}$. The initial air mass fraction of the mixture bubble is $Y_{o}=0.996$ as previously described. During the first negative pulsing, the bubble grows to $R_{b,\max}=7.64R_{\mathrm{bo}}$ and the bubble mass increases to $m_{b,\max}=2.59m_{\mathrm{bo}}$ with evaporation. During the bubble expansion, the bubble average pressure $p_{b,\mathrm{av}}$ decreases, as seen in Fig. 2c. However, $p_{b,\mathrm{av}}$ does not drop below the saturated vapor pressure $p_{v,\mathrm{sat}}$ at $T_{\infty }$ because of the phase-change vapor in the bubble. During the subsequent positive pulsing, the bubble rapidly shrinks and the bubble mass decreases. At the main bubble collapse near t=0.76μs, water vapor accounts for 5.5% of the mass in the bubble, which is consistent with the observation in Ref. [24]. Thereafter, the bubble mass increases and decreases with subsequent bubble rebounds and recollapses. The water vapor continues to condense immediately after the first rebound due to the still high bubble pressure [22]. As $p_{b,\mathrm{av}}$ significantly increases with bubble collapse, the bubble temperature $T_{b,\mathrm{av}}$ reaches $25.3T_{\infty }$. The cavitation threshold for bubble collapse temperature above 5000 K is obtained by increasing $p_{A}$ by 1kPa, resulting in 0.306MPa.

The phase-change mass flux *G* at the bubble surface is determined from $(q_{\mathrm{ls}}-q_{\mathrm{bs}})/h_{\mathrm{fg}}$, where $q_{\mathrm{ls}}$ and $q_{\mathrm{bs}}$ are the liquid and bubble side heat fluxes directly evaluated by temperature gradients. The heat fluxes $q_{\mathrm{ls}}$ and $q_{\mathrm{bs}}$ are determined by solving the full energy equations in the bubble and liquid domains, unlike most previous works [22], [23], [24], [25], [26], [27], [28], [29], [31] using the boundary layer approximations with adjusting factors.

**Fig. 3:**
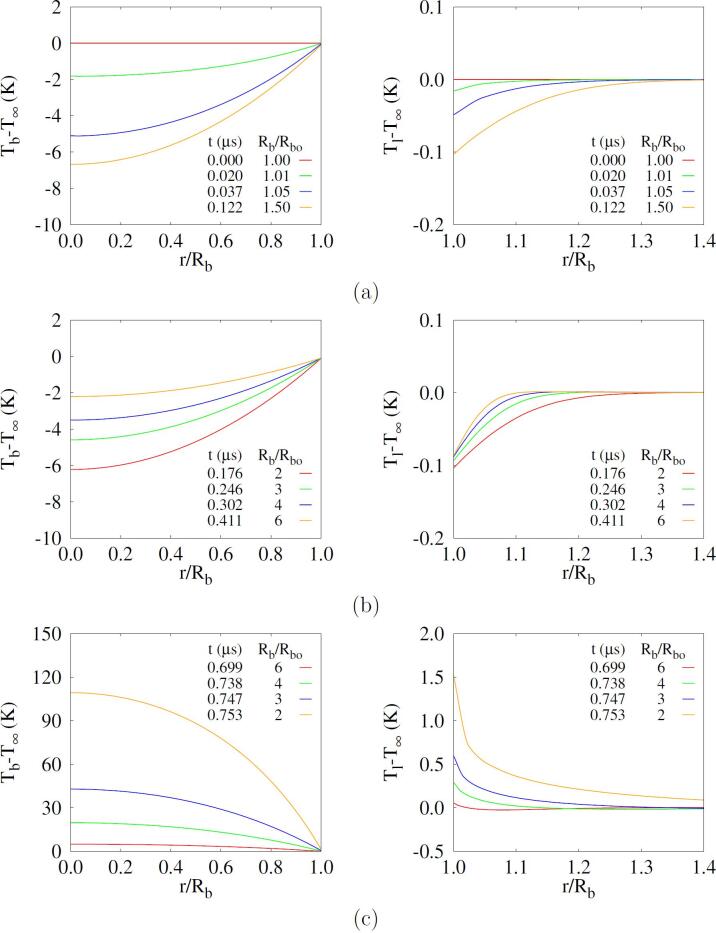
Temperature distributions inside the mixture bubble (left) and adjacent liquid (right) at $R_{\mathrm{bo}}=500\mathrm{nm},f=1\mathrm{MHz}$ and $p_{A}=0.35\mathrm{MPa}$ during: (a) the early expansion ($R_{b}⩽1.5R_{\mathrm{bo}}$), (b) expansion ($2R_{\mathrm{bo}}⩽R_{b}⩽6R_{\mathrm{bo}}$) and (c) shrinkage ($2R_{\mathrm{bo}}⩽R_{b}⩽6R_{\mathrm{bo}}$).

The computed local temperature distribution inside and outside the bubble is plotted in Fig. 3. During the early period of bubble expansion, the temperature inside the bubble decreases faster than the bubble surface temperature, while the liquid temperature drops slightly in the region of $r<1.4R_{b}$. During the bubble expansion period of t>0.176μs, the temperature in the bubble and liquid regions increases due to heat transfer from the surrounding liquid at $T_{\infty }$. As the bubble shrinks and collapses, the bubble temperature rises up rapidly, as seen in Fig. 3c. The temperature fields inside and outside the bubble are observed to vary more complex than predicted from the boundary layer approximation.

**Fig. 4:**
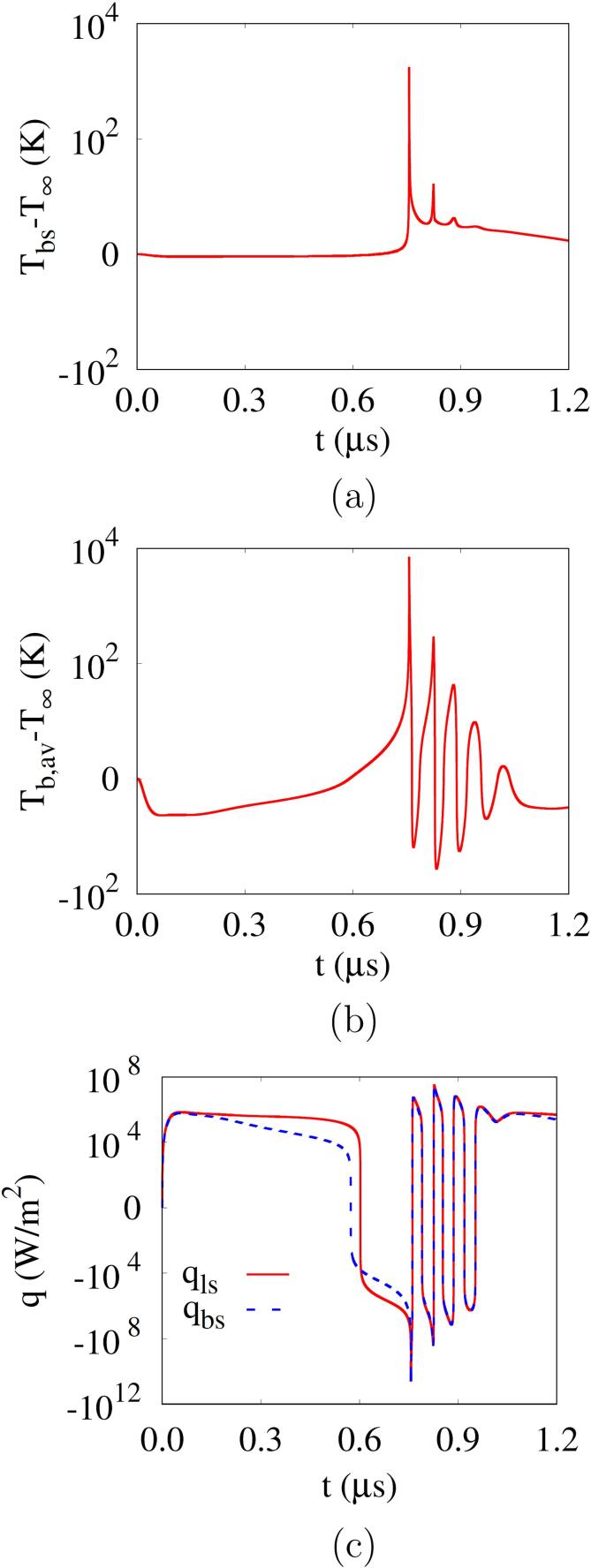
Bubble temperature and heat flux at $R_{\mathrm{bo}}=500\mathrm{nm},f=1\mathrm{MHz}$ and $p_{A}=0.35\mathrm{MPa}$: (a) bubble surface temperature, (b) bubble average temperature and (c) heat flux at the bubble surface.

Fig. 4 presents the bubble surface temperature $T_{\mathrm{bs}}$, the bubble average temperature $T_{b,\mathrm{av}}$ and the heat fluxes $q_{\mathrm{ls}}$ and $q_{\mathrm{bs}}$ obtained from the local temperature distributions. During the negative pulsing period, the bubble surface temperature $T_{\mathrm{bs}}$ is observed to remain near $T_{\infty }$ except for the bubble collapse periods, whereas the bubble average temperature $T_{b,\mathrm{av}}$ is slightly lower than $T_{\infty }$ and increases with the bubble shrinkage. During the bubble expansion, $q_{\mathrm{ls}}$ is higher than $q_{\mathrm{bs}}$, as the bubble temperature drops below $T_{\infty }$, which causes evaporation. Thereafter, as the bubble temperature rises rapidly with the positive pressure pulsing, $q_{\mathrm{ls}}$ is lower than $q_{\mathrm{bs}}$ and the vapor in the mixture bubble is condensed.

**Fig. 5:**
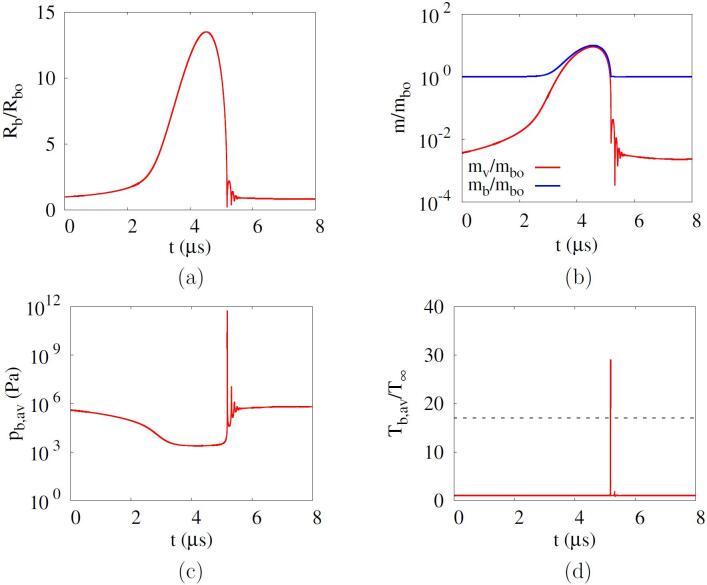
Acoustic cavitation of an air-vapor mixture bubble nucleus at $R_{\mathrm{bo}}=500\mathrm{nm},f=0.1\mathrm{MHz}$ and $p_{A}=0.202\mathrm{MPa}$: (a) bubble radius, (b) bubble mass, (c) bubble average pressure, (d) bubble average temperature. In the figure d, the dash line represents 5000K.

The effect of phase change on the acoustic cavitation at a lower frequency of f=0.1MHz and $p_{A}=0.202\mathrm{MPa}$ is plotted in Fig. 5. As the first negative pulsing period increases at the lower frequency, $R_{b,\max}$ and $m_{b,\max}$ significantly increase to $13.5R_{\mathrm{bo}}$ and $10.0m_{\mathrm{bo}}$, respectively. While the bubble expands, the mixture bubble pressure $p_{b,\mathrm{av}}$ remains near the water saturation pressure at $T_{\infty }$, as seen in Fig. 5c. This is coincident with the fact that cavitation is a phenomenon that occurs to maintain equilibrium with the saturation pressure when the pressure is lower than the saturation pressure [11], [33]. During the first bubble collapse, the bubble average temperature increases to a peak of $29.1T_{\infty }$ with a considerable water vapor accounting for 9.4% of the total bubble mass. This indicates that the phase change has a significant effect on both bubble growth and collapse dynamics during acoustic cavitation.

### 3.3 Blake threshold and acoustic cavitation threshold

The Blake threshold concept is useful to estimate the pressure required for explosive growth of a bubble, taking into account the quasi-static pressure and surface tension [14]. We briefly review the Blake threshold formulas for ideal and VDW gases. Assuming that the air-vapor mixture bubble is an ideal gas, bubble growth is an isothermal process and the vapor pressure $p_{v}$ is constant, the ambient liquid pressure is approximated as

(30) $$p_{l}=p_{b}-\frac{2\sigma }{R_{b}}=\rho_{\mathrm{ao}}{\left(\frac{R_{\mathrm{bo}}}{R_{b}}\right)}^{3}R_{g,a}T_{\infty }+p_{v}-\frac{2\sigma }{R_{b}}$$

where the initial air density $\rho_{\mathrm{ao}}$ is determined by $\rho_{\mathrm{ao}}=(p_{\infty }+2\sigma /R_{\mathrm{bo}}-p_{v})/R_{g,a}T_{\infty }$. The critical radius $R_{\mathrm{cr}}$ for unstable or explosive bubble growth is obtained by differentiating Eq. (30) with respect to $R_{b}$ [14], and the corresponding Blake threshold pressure $p_{B}$ is expressed as

(31) $$p_{B}=p_{\infty }-p_{l}=p_{\infty }+\frac{4\sigma }{3}\sqrt{\frac{2\sigma }{3R_{\mathrm{bo}}^{3}\rho_{\mathrm{ao}}R_{g,a}T_{\infty }}}-p_{v}$$

Using the EOS of VDW gas, Eq. (30) is rewritten as

(32) $$p_{l}=\frac{\rho_{\mathrm{bo}}{\left(R_{\mathrm{bo}}/R_{b}\right)}^{3}R_{g,b}T_{\infty }}{1-b_{b}\rho_{\mathrm{bo}}{\left(R_{\mathrm{bo}}/R_{b}\right)}^{3}}-a_{b}\rho_{\mathrm{bo}}^{2}{\left(\frac{R_{\mathrm{bo}}}{R_{b}}\right)}^{6}-\frac{2\sigma }{R_{b}}$$

and $R_{\mathrm{cr}}$ and $p_{B}$ are iteratively obtained with Eqs. (2), (3) for $a_{b}$ and $b_{b}$.

**Fig. 6:**
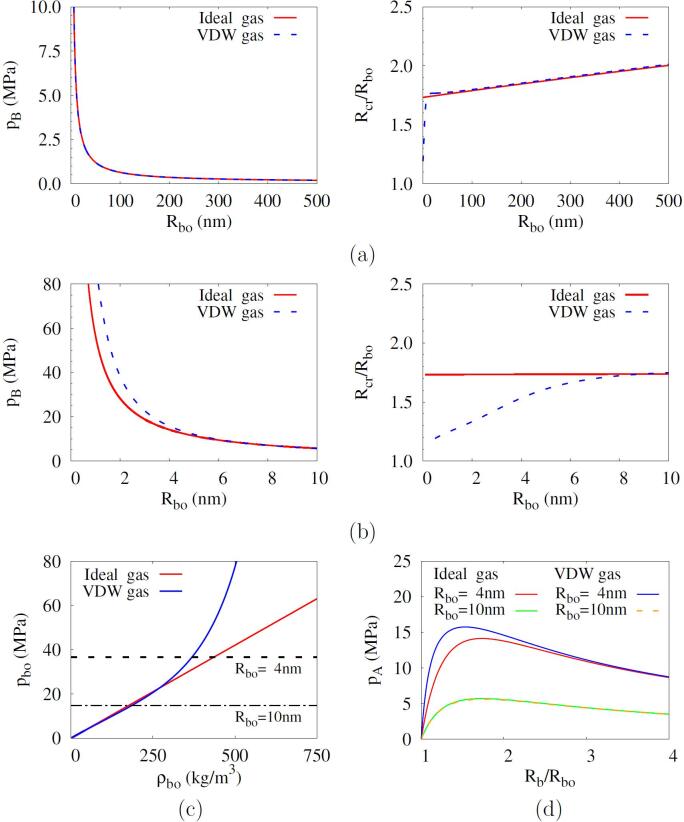
Blake threshold pressure and critical radius for the ideal and VDW gases: (a) in a wide range of $R_{\mathrm{bo}}⩽500\mathrm{nm}$ and (b) in an narrow range of $R_{\mathrm{bo}}⩽10\mathrm{nm}$; (c) $p_{\mathrm{bo}}$ versus $\rho_{\mathrm{bo}}$ and (d) $p_{A}$ versus $R_{b}/R_{\mathrm{bo}}$ for $R_{\mathrm{bo}}⩽10\mathrm{nm}$.

The predictions of $p_{B}$ and $R_{\mathrm{cr}}$ using the ideal and VDW gas equations for various initial radii keeping $p_{\infty }=1\mathrm{atm}$ and $T_{\infty }=293K$ are compared in Fig. 6. It is noted that the applied pressure is quasi-static with no frequency. The predictions for the VDW gas are almost identical to those for the ideal gas over a wide range of $R_{\mathrm{bo}}$. However, when $R_{\mathrm{bo}}$ decreases below 10nm, as in distilled water [9], [10], and the effect of surface tension becomes pronounced, the predictions for the VDW gas differ significantly from those for the ideal gas. Considering that the bubble is initially at $p_{\mathrm{bo}}=p_{\infty }+2\sigma /R_{\mathrm{bo}}$ and $T_{\mathrm{bo}}=T_{\infty }$, its initial density is evaluated directly from the ideal gas equation and iteratively from the VDW gas equation. As $R_{\mathrm{bo}}$ decreases below 10nm and $p_{\mathrm{bo}}$ increases, the initial density becomes different between the ideal and VDW gases, as seen in Fig. 6c. When the initial bubble radius is reduced to $R_{\mathrm{bo}}=4\mathrm{nm}$, the pressure $p_{A}$ required for bubble growth, evaluated as $p_{A}=p_{\infty }+2\sigma /R_{b}-p_{b}$ in the Blake threshold model, is significantly different between the ideal and VDW gases, as depicted in Fig. 6d. It is noted that the maximum value of $p_{A}$ corresponds to the Blake threshold $p_{B}$.

**Fig. 7:**
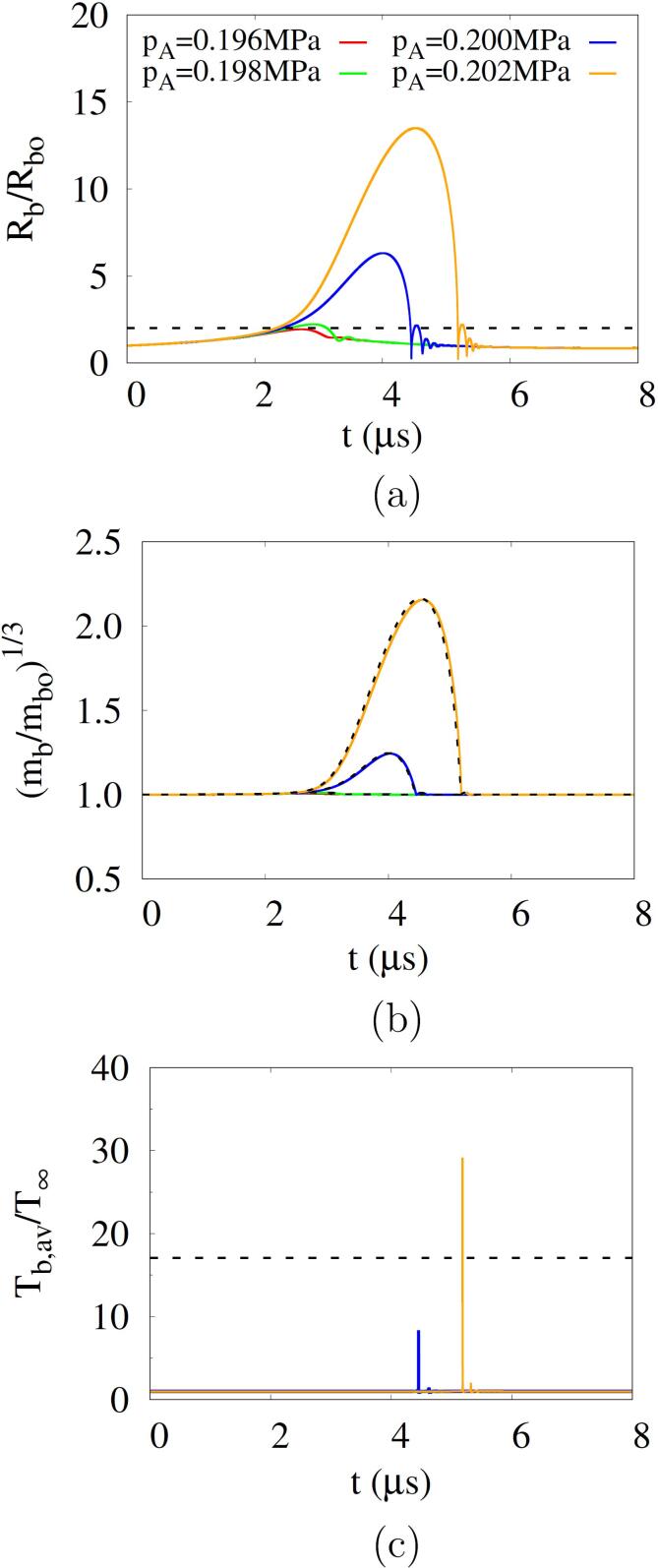
Influence of acoustic amplitude on acoustic cavitation at $R_{\mathrm{bo}}=500\mathrm{nm}$ and f=0.1MHz: (a) bubble radius, (b) bubble mass and (c) bubble average temperature. In the figures a, b and c, the dash lines represent $2R_{\mathrm{bo}}$, the approximated bubble mass and 5000K, respectively.

Fig. 7 shows the influence of $p_{A}$ on the acoustic cavitation at $R_{\mathrm{bo}}=500\mathrm{nm}$ and f=0.1MHz. For $R_{\mathrm{bo}}=500\mathrm{nm}$, the predicted Blake threshold $p_{B}$ is 0.196MPa and the corresponding critical radius $R_{\mathrm{cr}}$ is $2R_{\mathrm{bo}}$, which is coincident with the cavitation threshold criterion of $R_{b,\max}⩾2R_{\mathrm{bo}}$ [16], [17]. The curves of $R_{b}/R_{\mathrm{bo}}$ appears to be sensitive to the acoustic amplitude $p_{A}$ when $p_{A}$ is close to $p_{B}$. For $p_{A}=p_{B}$, the bubble grows to $R_{b,\max}=1.94R_{\mathrm{bo}}$, whereas for $p_{A}=0.198\mathrm{MPa}$, the bubble becomes larger than $R_{\mathrm{cr}}$ at t=2.54μs and then shrinks during the positive pulsing. As $p_{A}$ increases above 0.200 MPa, the bubble grows significantly and then collapses rapidly. The temporal variation of bubble mass $m_{b}$ also depends on $p_{A}$. The relation between $m_{b}/m_{\mathrm{bo}}$ and $R_{b}/R_{\mathrm{bo}}$ is expressed as

(33) $$\frac{m_{b}}{m_{\mathrm{bo}}}=\frac{\rho_{a}+\rho_{v}}{\rho_{\mathrm{ao}}+\rho_{\mathrm{vo}}}{\left(\frac{R_{b}}{R_{\mathrm{bo}}}\right)}^{3}$$

Using $\rho_{a}R_{b}^{3}=\rho_{\mathrm{ao}}R_{\mathrm{bo}}^{3},\rho_{v}\approx \rho_{\mathrm{vo}}$ and $\rho_{\mathrm{ao}}\gg \rho_{\mathrm{vo}}$, Eq. (33) can be approximated as

(34) $$\frac{m_{b}}{m_{\mathrm{bo}}}\approx 1+\frac{\rho_{\mathrm{vo}}}{\rho_{\mathrm{ao}}}{\left(\frac{R_{b}}{R_{\mathrm{bo}}}\right)}^{3}$$

The approximations match well with the numerical predictions (Fig. 7b). When the bubble collapses strongly for $p_{A}⩾0.200\mathrm{MPa}$, sharp peaks appear in the bubble temperature (Fig. 7c). The peak values increase with increasing $p_{A}$ and exceed the cavitation criterion of $T_{b,\max}=5000K$ for $p_{A}⩾0.201\mathrm{MPa}$. At the low acoustic frequency of 0.1MHz, the cavitation threshold is observed to be close to the Blake threshold. However, the Blake threshold concept, which does not take into account the temporal influence of acoustic frequency, can causes a deviation from the acoustic cavitation threshold at higher frequencies. The influence of acoustic frequency will be investigated in the next section.

### 3.4 Effect of acoustic frequency

**Fig. 8:**
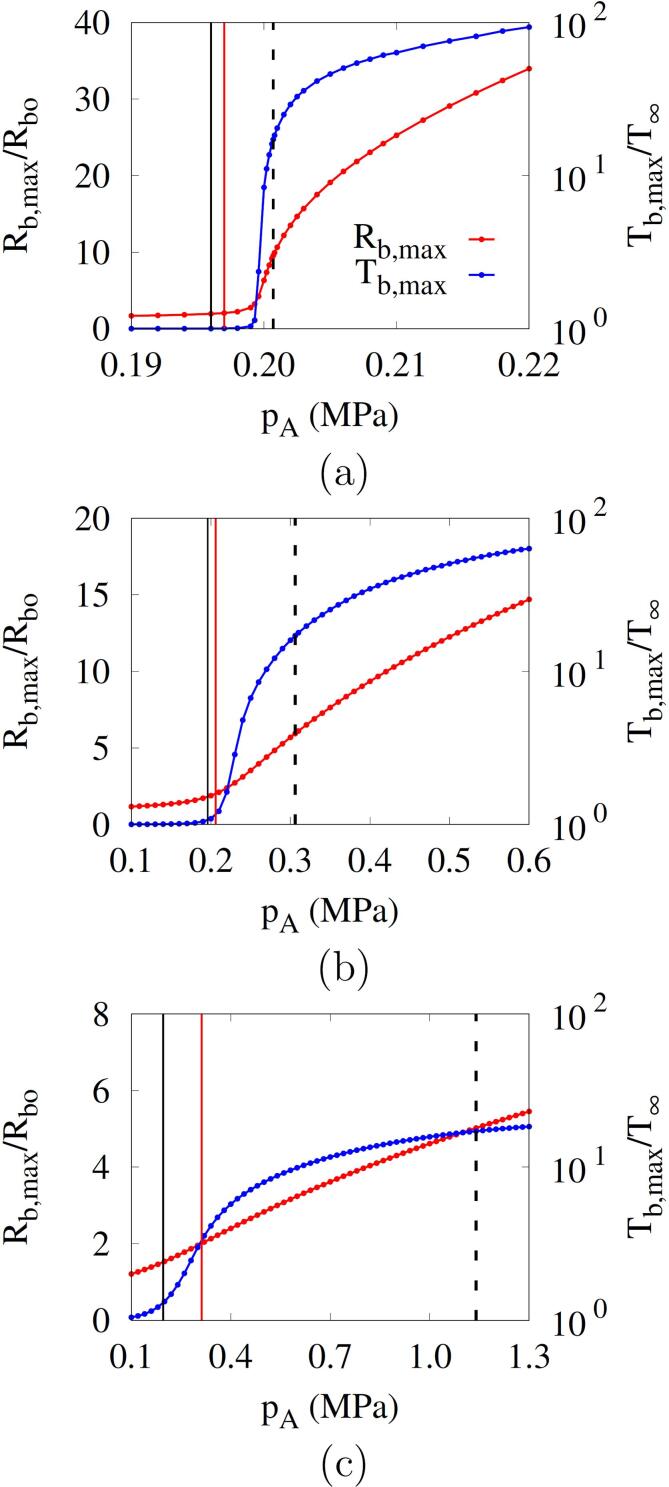
Effect of acoustic amplitude on the maximum bubble radius and temperature during acoustic cavitation at $R_{\mathrm{bo}}=500\mathrm{nm}$ and different frequencies: (a) f=0.1MHz, (b) f=1MHz and (d) f=5MHz. The vertical black solid line represents $p_{B}$, and the red solid and black dash lines represent $p_{2R_{\mathrm{bo}}}$ and $p_{5000K}$, respectively.

Fig. 8 presents the effects of *f* and $p_{A}$ on the maximum bubble radius and temperature. The vertical black solid line denotes the Blake threshold $p_{B}$ whereas the red solid and black dash lines denote the minimum acoustic amplitudes $p_{2R_{\mathrm{bo}}}$ and $p_{5000K}$ for $R_{b,\max}⩾2R_{\mathrm{bo}}$ and $T_{b,\max}⩾5000K$, respectively. The results are obtained only during the period prior to the first bubble rebound to remove the uncontrolled influences of subsequent bubble collapses and rebounds. For $p_{A}⩽p_{B}(=0.196\mathrm{MPa})$, $R_{b,\max}$ is less than $2R_{\mathrm{bo}}(=R_{\mathrm{cr}})$ regardless of the acoustic frequency. For a low frequency of f=0.1MHz (Fig. 8a), the bubble begins to grow abruptly at $p_{A}=0.2\mathrm{MPa}$ and the thresholds $p_{2R_{\mathrm{bo}}}$ and $p_{5000K}$ are close to the Blake threshold $p_{B}$. However as the frequency increases to 1 MHz and 5 MHz, the increases of $R_{b,\max}$ and $m_{b,\max}$ with $p_{A}$ are reduced because the negative pulsing period decreases. The threshold $p_{2R_{\mathrm{bo}}}$ based on $R_{b,\max}⩾2R_{\mathrm{bo}}$ increases to 0.206MPa for f=1MHz and 0.312MPa for f=5MHz as depicted in Fig. 8b and c. The threshold $p_{5000K}$ based on $T_{b,\max}⩾5000K$ increases to 0.306MPa and 1.140MPa for f=1MHz and 5MHz, respectively. This indicates that as *f* increases, the cavitation thresholds $p_{2R_{\mathrm{bo}}}$ and $p_{5000K}$ become larger than the Blake threshold $p_{B}$, which was derived without taking into account the temporal influence of acoustic frequency. The threshold difference $p_{5000K}-p_{2R_{\mathrm{bo}}}$ also increases with the acoustic frequency.

### 3.5 Effect of bubble nucleus size

**Fig. 9:**
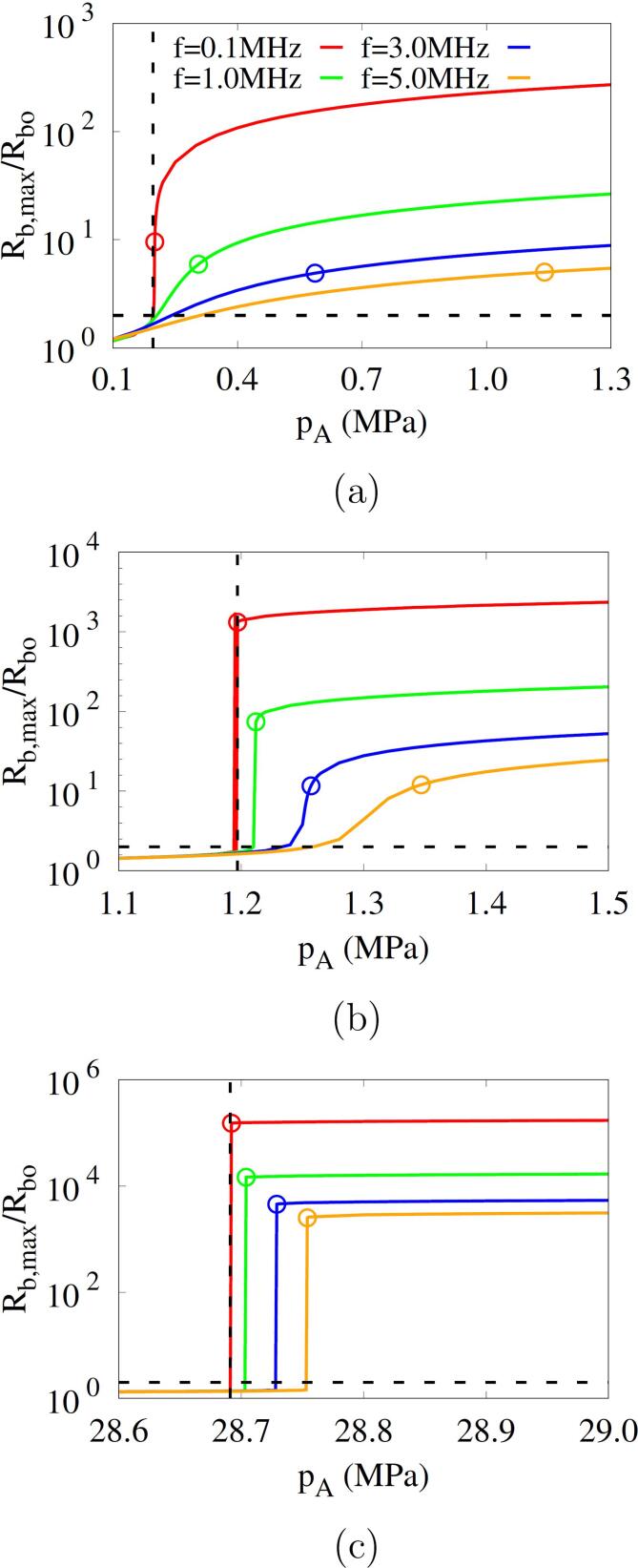
Combined effects of acoustic frequency and amplitude on the bubble maximum radius during acoustic cavitation at different bubble nuclei: (a) $R_{\mathrm{bo}}=500\mathrm{nm}$, (b) $R_{\mathrm{bo}}=50\mathrm{nm}$ and (c) $R_{\mathrm{bo}}=2.5\mathrm{nm}$. In the figures, the horizontal and vertical dash lines represent $2R_{\mathrm{bo}}$ and $p_{B}$, respectively, and the circle symbols represent the result of $p_{5000K}$.

Fig. 9 shows the combined effects of bubble nucleus size and acoustic amplitude and frequency on the bubble growth $R_{b,\max}$ in acoustic cavitation. For f=0.1MHz, the thresholds $p_{2R_{\mathrm{bo}}}$ and $p_{5000K}$ are observed to be close to the Blake threshold $p_{B}$ regardless of the bubble nucleus size. For $R_{\mathrm{bo}}=500\mathrm{nm}$, as seen in Fig. 9a, $R_{b,\max}$ at f=0.1MHz increases abruptly near $p_{A}=p_{B}$, and as *f* increases and the first negative pulsing period decreases, the variation of $R_{b,\max}$ slows and the thresholds increase. As the nucleus size is reduced to $R_{\mathrm{bo}}=50\mathrm{nm}$ and $R_{\mathrm{bo}}=2.5\mathrm{nm}$ (Fig. 9b and c), the thresholds $p_{2R_{\mathrm{bo}}}$ and $p_{5000K}$ as well as $p_{B}$ increase significantly. The change of $R_{b,\max}$ for $p_{A}$ becomes steep over a wide range of acoustic frequencies. The thresholds $p_{2R_{\mathrm{bo}}}$ and $p_{5000K}$ are closer to $p_{B}$ as $R_{\mathrm{bo}}$ decreases.

**Fig. 10:**
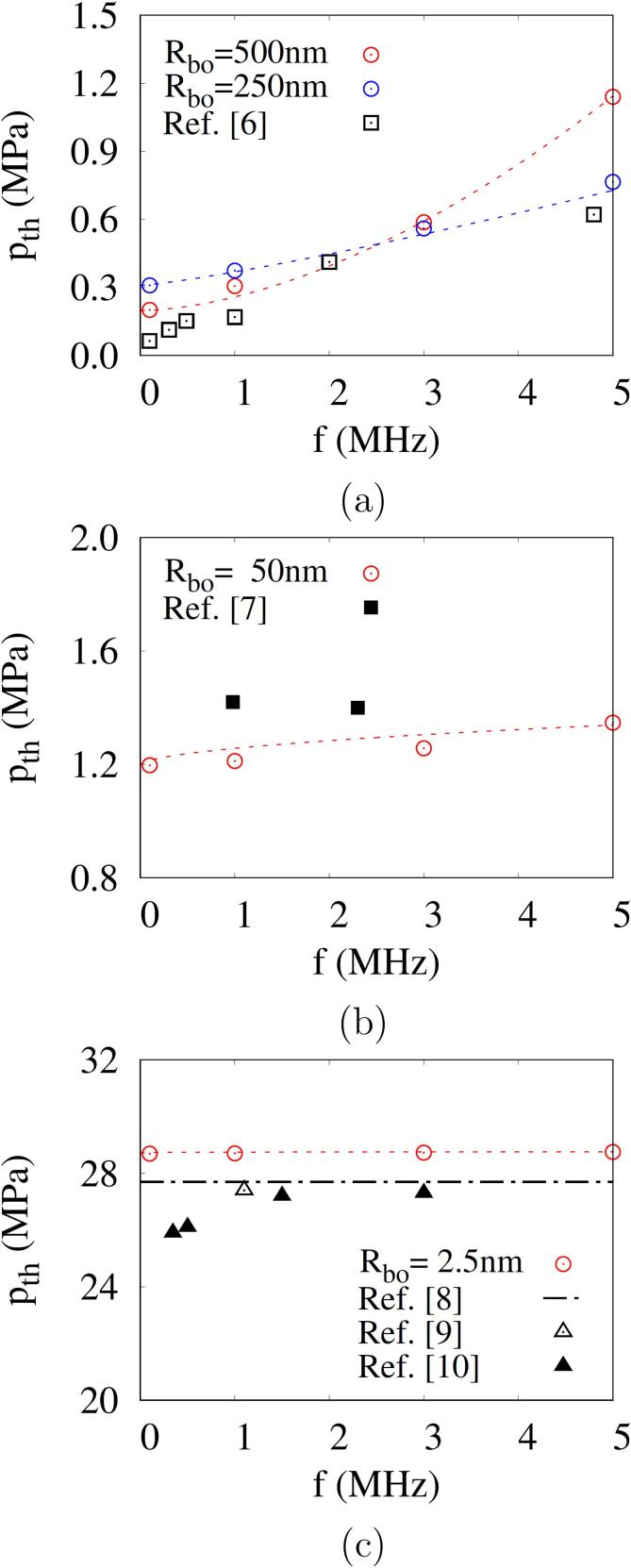
Predicted cavitation thresholds versus acoustic frequencies at different bubble nucleus radii: (a) $250\mathrm{nm}⩽R_{\mathrm{bo}}⩽500\mathrm{nm}$, (b) $R_{\mathrm{bo}}=50\mathrm{nm}$ and (c) $R_{\mathrm{bo}}=2.5\mathrm{nm}$. The dash lines are the fitted curves from the numerical results of $p_{5000K}$ (circle symbols), and the black symbols are the experimental data in the previous works.

In Fig. 10, the present predictions of cavitation threshold $p_{5000K}$ (or $p_{\mathrm{th}}$) are compared with experimental data reported in the literature [6], [7], [8], [9], [10]. The experimental data were obtained using air-saturated or degassed water. The acoustic cavitation thresholds measured in air-saturated water are 0.02-0.62MPa for 0.02MHz⩽f⩽4.8MHz [6]. The experimental data are comparable to the numerical prediction for a relatively large bubble nucleus with $R_{\mathrm{bo}}⩾250\mathrm{nm}$, as seen in Fig. 10a. The bubble nuclei in gas-saturated water are expected to easily grow to larger sizes due to coalescence during acoustic pulsing periods [40], and the cavitation threshold is observed to decrease as the air saturation in water increases [41]. For degassed water [7], [8], [9], [10], the cavitation threshold increases as depicted in Fig. 10b and c. The present predictions for $R_{\mathrm{bo}}=50\mathrm{nm}$ can be compared with the experimental data of Atchley et al. [7] for f=0.98MHz and 2.3MHz using less than two acoustic pulses. The present predictions for $R_{\mathrm{bo}}=2.5\mathrm{nm}$ are also comparable to the experimental data of Refs. [9], [10], in a range of $25.9\mathrm{MPa}⩽p_{\mathrm{th}}⩽27.4\mathrm{MPa}$ for 0.35MHz⩽f⩽3MHz. The effect of acoustic frequency on the cavitation threshold weakens as $R_{\mathrm{bo}}$ decreases. This can be explained by considering the scales of inertia, surface tension and viscous stress that affect the bubble pressure, as expressed in Eq. (22). Selecting $R_{\mathrm{bo}}$ and 1/f as length and time scales, which is based on the observation in Fig. 9 that the cavitation threshold $p_{\mathrm{th}}$ is close to the minimum acoustic amplitude for $R_{b,\max}⩾5R_{\mathrm{bo}}$, the scales of inertia, surface tension and viscous stress can be estimated as $\rho_{l}R_{\mathrm{bo}}^{2}f^{2},\sigma /R_{\mathrm{bo}}$ and $\mu_{l}f$, respectively. Therefore, as $R_{\mathrm{bo}}$ decreases, the surface tension effect is dominant and the frequency effect becomes relatively weak.

The predicted thresholds are fitted within the root-mean-square error of 0.03 MPa as

(35) $$p_{\mathrm{th}}=p_{B}+0.06f^{0.075R_{\mathrm{bo}}^{0.5}}$$

where $p_{\mathrm{th}},f$ and $R_{\mathrm{bo}}$ are in MPa,MHz and *nm*, respectively. This fitted equation is applicable to acoustic cavitation in water with a wide range of cavitation thresholds (0.2–30 MPa) reported in the literature, as seen in Fig. 10. Eq. (35) is not very useful unless the bubble nucleus size is known. However, the equation is effective in quantifying the combined effect of bubble nucleus size and acoustic frequency on the difference between the cavitation threshold and the Blake threshold.

## 4 Conclusion

A numerical model for acoustic cavitation threshold in water was developed by coupling the Rayleigh-Plesset or Keller-Miksis equation with the energy equation for the bubble and liquid regions and directly evaluating the phase-change rate from the liquid and bubble side temperature gradients. The numerical model was applied to elucidate acoustic cavitation in water with a wide range of cavitation thresholds (0.02–30 MPa) reported in the literature. The numerical results showed that the temperature distribution inside and outside the bubble varies more complex than predicted from the boundary layer approximation. The phase-change vapor was observed to have a significant effect on bubble growth and collapse dynamics during acoustic cavitation. As the bubble nucleus size increases and the acoustic frequency increases, the cavitation threshold increases beyond the Blake threshold, which was developed without taking into account the temporal influence of acoustic frequency. The predicted thresholds were fitted as a function of bubble nucleus size and acoustic frequency and could be applied to acoustic cavitation in water with a wide range of threshold data reported in the literature.

## CRediT authorship contribution statement

**Seongjin Hong:** Writing - original draft, Conceptualization, Software. **Gihun Son:** Writing - review & editing, Conceptualization, Methodology, Supervision.

## Declaration of Competing Interest

The authors declare that they have no known competing financial interests or personal relationships that could have appeared to influence the work reported in this paper.
